# Early Oral Immunotherapy with Pasteurized Egg White in Children Younger than Two Years with IgE-Mediated Egg Allergy: A Prospective Study with Historical Controls

**DOI:** 10.3390/children13060810

**Published:** 2026-06-12

**Authors:** Silvia Karina Carrión Sari, Luis Martínez-Lostao, Carlos Colás Sanz, David Jerves Donoso, Diego Fernández-Lázaro, María Teresa Sobrevia Elfau

**Affiliations:** 1 Allergy Unit, University Healthcare Complex of Soria (CAUSO), Castilla y León Health System, 42005 Soria, Spain; karinacarrions@hotmail.com; 2 Immunology Department, Lozano Blesa University Clinical Hospital, 50009 Zaragoza, Spain; lmartinezlos@salud.aragon.es; 3 Aragon Health Research Institute (IIS Aragón), 50009 Zaragoza, Spain; ccolas@unizar.es (C.C.S.); materesas@hotmail.com (M.T.S.E.); 4 Faculty of Medicine, University of Zaragoza, 50009 Zaragoza, Spain; 5 Allergy Department, Lozano Blesa University Clinical Hospital, 50009 Zaragoza, Spain; 6 Pulmonology Department, University Healthcare Complex of Soria (CAUSO), Castilla y León Health System, 42005 Soria, Spain; davidjerves@hotmail.es; 7 Neurobiology Research Group, Faculty of Medicine, University of Valladolid, 47005 Valladolid, Spain; 8 Histology Area, Faculty of Health Sciences, Campus of Soria, University of Valladolid, 42004 Soria, Spain; 9 Consolidated Research Group ENSADE (Envejecimiento, Neurociencia, Salud y Desarrollo), León Biosanitary Research Institute (IBIOLEÓN), 24071 León, Spain

**Keywords:** desensitization, egg allergy, food allergy, immune modulation, infants, oral immunotherapy

## Abstract

**Highlights:**

**What are the main findings?**

- Early oral immunotherapy (OIT) with pasteurized egg white in children younger than two years achieved high desensitization rates, with 93.5% of treated patients able to consume all egg preparations, including raw egg, after six months of follow-up.
- Early OIT was associated with favorable short-term safety outcomes and significant immunological changes, including reductions in skin prick test wheal diameters, specific IgE levels, and specific IgE/total IgE ratios, together with increased specific IgG4 levels.

**What are the implications of the main findings?**

- Early active intervention with OIT during infancy may represent a feasible alternative to strict avoidance strategies and may help reduce severe reactions associated with accidental egg exposure during a critical period of immune development.
- The favorable efficacy and safety profile observed in very young children supports consideration of OIT as an early therapeutic option in specialized allergy centers, with potential benefits for dietary diversification and quality of life.

**Abstract:**

Background: Egg allergy is one of the most common food allergies in early childhood and is traditionally managed through strict avoidance diets, which may negatively affect nutrition and quality of life. Early oral immunotherapy (OIT) may represent an alternative therapeutic strategy; however, controlled studies in children younger than two years remain limited. Methods: We conducted a prospective observational study using historical controls. Thirty-one children younger than two years with IgE-mediated egg allergy underwent OIT using pasteurized liquid egg white (maximum dose: 30 mL; 3300 mg protein). Twelve children managed with an avoidance diet served as the historical control group. Outcomes included desensitization rates, adverse reactions, and longitudinal changes in skin prick test (SPT) wheal diameters, serum-specific IgE (sIgE), specific IgG4 (sIgG4), and sIgE/total IgE ratios. Results: At six months, 29/31 children (93.5%) in the OIT group did not experience allergic reactions after ingestion of any egg preparation, compared with none in the historical control group (*p* < 0.001). In the control group, 7/12 children (58.3%) continued to react to less-cooked egg preparations, whereas 5/12 (41.7%) remained reactive to all forms of eggs. During the induction phase, 24/31 OIT-treated children (77.4%) experienced mild adverse reactions, predominantly isolated cutaneous or gastrointestinal symptoms, and no patient required intramuscular adrenaline administration. In contrast, allergic reactions occurred in 11/12 controls, including anaphylaxis in 6/12 (50.0%) patients (*p* = 0.0301). The OIT group demonstrated significant reductions in SPT wheal diameters, sIgE levels, and sIgE/total IgE ratios (all *p* < 0.001), accompanied by increased sIgG4 levels. Conclusions: Early OIT with pasteurized egg white in children younger than two years with IgE-mediated egg allergy was associated with high desensitization rates, favorable short-term safety outcomes, and significant immunological changes. These findings support the potential role of early active intervention as an alternative to exclusive avoidance strategies in infants with egg allergy.

## 1. Introduction

Patients with food allergy experience a significant deterioration in quality of life, affecting both children and their families, largely because of exclusion diets and the constant fear of accidental reactions [1]. When the offending food is highly nutritious, concern extends beyond accidental exposure to the potential risk of impaired growth and nutritional deficiencies resulting from strict avoidance diets [2].

Hen’s egg allergy is particularly prevalent during the first years of life and remains one of the most common food allergies in early childhood [3]. Current estimates place the prevalence of egg allergy in children under two years of age between 0.5% and 1.8% [4]. As an alternative to strict avoidance diets, oral immunotherapy (OIT) has emerged in recent years as a therapeutic strategy aimed at facilitating the controlled introduction of allergenic foods and promoting desensitization [4]. Recently, Elkan et al. [5] reported that OIT may improve long-term growth trajectories in food-allergic children, particularly among younger patients with egg allergy.

Eggs are an important source of high biological value protein and essential micronutrients. A medium-sized egg contains approximately 6.4 g of protein, 4.6 g of total fat (including around 1.7 g of monounsaturated fatty acids), and about 66 kcal [6]. In populations with increased nutritional requirements—such as infants, children, pregnant women, and athletes—eggs provide several key nutrients, including vitamin D, choline, folate, iodine, phosphorus, selenium, vitamin A, vitamin B12, riboflavin, biotin, and pantothenic acid, many of which are commonly underconsumed in habitual diets [7].

IgE-mediated egg allergy manifests with a spectrum of clinical symptoms, ranging from mild cutaneous reactions (perioral erythema or urticaria) to multi-organ involvement in anaphylaxis [8]. In clinical practice, diagnosis is based on a compatible clinical history together with skin prick test (SPT), specific IgE (sIgE) determination and, when necessary, an oral food challenge (OFC) to confirm the diagnosis [9].

Once a diagnosis of food allergy has been established, management traditionally relies on allergen avoidance, dietary counseling, and, more recently, OIT [9]. Avoidance strategies are particularly challenging in egg allergy because egg proteins are ubiquitous in processed and prepared foods, increasing the risk of accidental exposures and potentially severe reactions [10,11].

There is growing interest in early immunotherapy and its potential therapeutic benefit. During early infancy, the maturation of the IgE response is delayed or immature, representing a period of increased immune plasticity that may favor effective intervention through early treatment, thereby potentially preventing the development of persistent food allergy [12].

We therefore hypothesized that oral immunotherapy in children under two years of age with egg protein allergy effectively reintegrates egg into their regular diet, in contrast to untreated allergic patients. Accordingly, the objective of this study was to compare the clinical efficacy, immunological response, and safety profile of oral immunotherapy with pasteurized egg white versus an avoidance diet in children under two years of age with IgE-mediated egg allergy.

## 2. Material and Methods

### 2.1. Study Design

We conducted a prospective, analytical, observational study using historical controls. The study was reported in accordance with the Strengthening the Reporting of Observational Studies in Epidemiology (STROBE) statement [13]. The completed STROBE checklist is provided in Supplementary File S1.

The prospective OIT protocol was approved by the Research Ethics Committee of the Autonomous Community of Aragon (CEICA; protocol code PI19/315; Supplementary File S2). In addition, the retrospective observational study involving historical controls was reviewed and favorably acknowledged by the Drug Research Ethics Committee of Burgos and Soria (CEIm Burgos-Soria; reference number 2880; Supplementary File S3).

Written informed consent was obtained from both parents and/or legal guardians of all children included in the OIT group. For the historical control group, the Ethics Committee approved the use of previously collected clinical data based on the informed consent obtained during routine clinical care, considering the retrospective nature of the study and the impracticability of obtaining renewed consent years after the original clinical evaluation.

The study was not pre-registered in a clinical trial registry because it is an observational study with historical controls; however, it adheres to STROBE guidelines [13].

### 2.2. Setting and Participants

Children with confirmed IgE-mediated hen’s egg allergy who were evaluated at the Allergology Department, University Clinical Hospital “Lozano Blesa”, Zaragoza, Spain, between 2019 and 2023 were consecutively recruited into the OIT group. Historical controls were selected from children evaluated at the Pediatric and Allergology Departments, University Hospital “Santa Bárbara”, Soria, Spain, between 2015 and 2022, where egg OIT was not available during the study period. Given the restricted age range of the target population (<2 years), all children who fulfilled the eligibility criteria and had a follow-up assessment six months after diagnosis were consecutively included in the control group to maximize sample size and reduce selection bias.

Participant selection, follow-up, and final analysis are summarized in the STROBE flow diagram (Figure S1).

### 2.3. Inclusion Criteria

Children younger than 2 years of age with confirmed IgE-mediated hen’s egg allergy were eligible for inclusion. Diagnosis was established based on the following criteria: (i) a documented history of an immediate allergic reaction occurring within 1 h after egg ingestion; (ii) positive sensitization demonstrated by serum sIgE levels > 0.35 kU/L and/or a positive SPT, defined as a wheal diameter ≥ 3 mm above the negative control, to at least one egg component (egg white, egg yolk, ovalbumin [OVA], or ovomucoid [OVM]); and (iii) a positive OFC with boiled egg [9]. Baseline OFC was not performed in children with a documented history of anaphylaxis according to Sampson’s criteria [14], or in those presenting with two or more immediate reactions within the preceding three months, due to safety considerations.

### 2.4. Oral Immunotherapy Group (Active Group)

A total of 31 children underwent OIT using pasteurized liquid egg white (Huevos Guillén S.L.^®^, Paterna, Valencia, Spain; 11 g protein/100 g egg white). The protocol was adapted from the pasteurized egg white OIT protocol used in the multicenter SEICAP study reported by Martín-Muñoz et al. [15]. OIT consisted of two sequential phases: induction and maintenance.

During the induction phase, patients underwent weekly supervised dose escalations in the hospital setting (Supporting Information Table S1), beginning with 0.1 mL of pasteurized egg white (approximately 11 mg of egg protein) and progressively increasing to a target dose of 30 mL (approximately 3300 mg of egg protein, equivalent to one medium-sized egg). Between hospital visits, the same dose administered during the previous hospital visit was given daily at home under parental supervision.

In the maintenance phase, once the target dose had been achieved, patients received 30 mL of pasteurized egg white on alternate days for 15 days. Subsequently, they were instructed to ingest at least one whole egg, prepared in different forms including less-cooked preparations (e.g., mayonnaise or mousse), three times weekly for six months.

At the completion of the induction phase (T2), an OFC with raw egg was performed to assess complete desensitization.

### 2.5. Control Group (Historical Avoidance Group)

The control group comprised 12 children with confirmed IgE-mediated hen’s egg allergy who were managed with a strict egg avoidance diet according to standard clinical practice. No OIT was administered during the study period. Clinical and laboratory data were retrospectively obtained from medical records at baseline diagnosis (T1) and at the six-month follow-up visit (T3). Measurements of specific IgG4 (sIgG4) were not available in the control group because these determinations were not routinely performed in clinical practice.

### 2.6. Clinical and Immunological Assessments

Clinical and immunological evaluations were performed at predefined study time points. Baseline assessment was defined as T1 for both groups. In the OIT group, evaluations were additionally performed at the end of the induction phase (T2) and six months later (T3). In the historical control group, follow-up evaluation was performed six months after diagnosis and was considered equivalent to T3 for comparative analyses.

The following parameters were assessed:

- SPTs: Wheal diameter (mm) to whole egg, egg white, egg yolk, OVA, and OVM.
- sIgE: Serum levels (kU/L) against the same egg components.
- Total IgE: Serum total IgE concentration (kU/L).
- sIgE/total IgE ratio: Calculated for each allergenic component.
- sIgG4: Serum levels (mg/L) against egg white, egg yolk, OVA, and OVM. These measurements were available only in the OIT group, as they were not routinely determined in the historical control cohort.

Adverse reactions occurring during both the induction and maintenance phases were prospectively recorded. For descriptive purposes, reaction severity was categorized as mild, moderate, or severe according to the Clark Severity Grading System [16]. Anaphylactic reactions were defined according to the criteria proposed by Sampson et al. [14].

**Figure 1:**
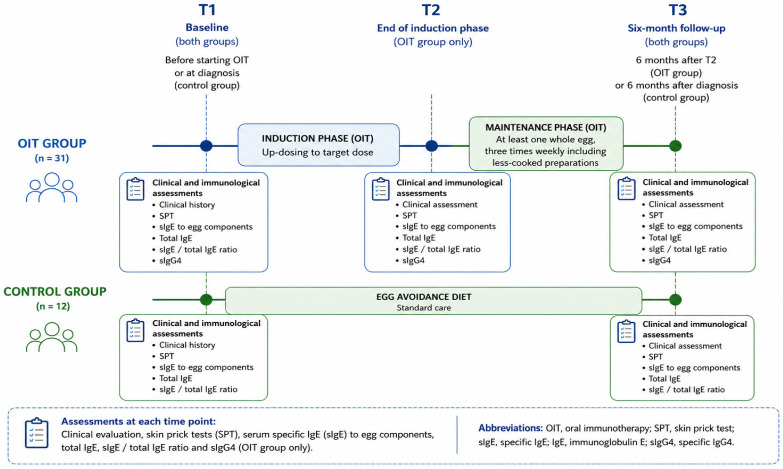
Study timeline and assessments. Clinical and immunological evaluations were conducted at predefined time points in the oral immunotherapy (OIT) and historical control groups. Baseline assessment (T1) was performed before initiation of OIT or at diagnosis in the control group. In the OIT group, additional evaluations were conducted at the end of the induction phase (T2) and six months later during the maintenance phase (T3). In the historical control group, follow-up evaluation was performed six months after diagnosis and considered equivalent to T3 for comparative analyses. Clinical and immunological assessments included clinical evaluation, skin prick tests (SPT), serum-specific IgE (sIgE), total IgE, the sIgE/total IgE ratio, and specific IgG4 (sIgG4) measurements (OIT group only). *Abbreviations*: IgE, immunoglobulin E; OIT, oral immunotherapy; sIgE, specific IgE; sIgG4, specific IgG4; SPT, skin prick test.

The study timeline, intervention phases, and assessment time points for both groups are summarized in Figure 1.

### 2.7. Outcome Measures

The primary outcome was the achievement of complete desensitization to egg at the end of the induction phase, confirmed by a negative OFC with raw egg.

Secondary outcomes included:

(i) changes in SPT wheal diameter;
(ii) changes in serum immunological parameters, including sIgE, total IgE, sIgE/total IgE ratio, and sIgG4.
(iii) frequency and severity of adverse reactions during the induction and maintenance phases.
(iv) persistence of the desensitized state to different egg preparations at the six-month follow-up visit (T3).

### 2.8. Statistical Analysis

Given the relatively small sample size (<50 patients per group), normality of quantitative variables was assessed using the Shapiro–Wilk test. Quantitative variables were expressed as median and range or interquartile range (IQR), as appropriate. Between-group comparisons were performed using the Mann–Whitney U test. Within-group longitudinal comparisons (T1 vs. T3) were analyzed using the Wilcoxon signed-rank test.

Categorical variables were summarized as absolute frequencies and percentages and compared using the chi-square test or Fisher’s exact test, as appropriate. All statistical tests were two-sided, and a *p*-value < 0.05 was considered statistically significant.

Statistical analyses were performed using SAS software version 9.4 (SAS Institute, Cary, NC, USA) and IBM SPSS Statistics version 25 (IBM Corp., Armonk, NY, USA).

## 3. Results

### 3.1. Baseline Clinical Characteristics

A total of 31 children were included in the OIT group and 12 in the historical control group. The median age at diagnosis was 11 months (range: 6–24 months) in the OIT group and 12.5 months (range: 6–23 months) in the control group. No statistically significant differences were observed between groups regarding sex distribution, personal history of atopic dermatitis, family history of allergic disease, or sensitization to other foods (Table 1).

At baseline (T1), SPT wheal diameters for egg white, OVA, and OVM were significantly higher in the OIT group compared with controls, whereas no significant differences were observed for egg yolk SPT or serum total IgE and sIgE levels to the evaluated egg components (Table 1). The clinical characteristics of the reactions leading to diagnosis were comparable between groups and are summarized in Table 1. Cutaneous manifestations were the most frequent presentation in both cohorts (Table 1), whereas anaphylaxis was observed in 43.3% of patients in the OIT group and 16.7% of controls, without statistically significant differences.

### 3.2. Characteristics of the Reactions Leading to Diagnosis

In the OIT group, 19 patients (61.3%) experienced reactions after ingestion of boiled egg, whereas 12 (38.7%) reacted to undercooked egg preparations. In the historical control group, reactions to boiled egg were observed in 5 patients (41.7%), while 7 (58.3%) reacted to undercooked preparations.

Regarding the clinical manifestations leading to diagnosis, cutaneous reactions were the most frequent presentation in both groups. In the OIT group, 13 patients (43.3%) presented with cutaneous manifestations, including perioral erythema, angioedema, and/or urticaria; 4 (13.3%) experienced isolated gastrointestinal symptoms (vomiting); and 13 (43.3%) experienced anaphylactic reactions.

In the control group, 10 patients (83.3%) presented cutaneous manifestations and 2 (16.7%) experienced anaphylactic reactions. No statistically significant differences were observed between groups regarding the clinical presentation of reactions at diagnosis.

### 3.3. Baseline Oral Food Challenge

#### 3.3.1. Oral Immunotherapy Group

In the OIT group, a baseline OFC with boiled egg was performed in 8 patients (25.8%), all of whom showed a positive result. Baseline OFC was not performed in the remaining patients because 8 had a documented history of anaphylaxis, 11 had experienced more than two immediate cutaneous reactions, and 3 had presented more than two gastrointestinal reactions within the preceding three months. In these cases, the combination of a compatible clinical history and positive allergy testing was considered sufficient to confirm the diagnosis of egg allergy.

The clinical manifestations observed during the OFC included urticaria and/or angioedema in 57.1% of patients, isolated vomiting in 14.3%, and anaphylactic reactions in 28.6%. The median dose of boiled egg triggering symptoms was 8.7 g (range: 1.56–25 g). The mean age at the time of OFC was 12.5 months (range: 8.7–20.3 months).

#### 3.3.2. Baseline Assessment of the Historical Control Group

In the historical control group, baseline OFC was performed in all patients who had not experienced reactions during the preceding three months (*n* = 8/12, 66.7%). The median eliciting dose was 4.9 g of boiled egg (range: 3.25–12.5 g), and the mean age at OFC was 12.9 months (standard deviation [SD]: 2.58).

During the OFC, among these 8 patients, 2 (25.0%) developed anaphylaxis, whereas 6 (75.0%) presented urticaria and/or angioedema.

No statistically significant differences were observed between groups regarding the eliciting dose (*p* = 0.805) or age at OFC performance (*p* = 0.408).

### 3.4. Oral Immunotherapy with Pasteurized Egg White

As previously reported by our group, 30 of 31 patients successfully completed the induction phase and achieved complete desensitization, confirmed by a negative OFC with raw egg [17]. One patient discontinued treatment for non-medical reasons after reaching a dose of 22 mL of pasteurized egg white.

Overall, 24 patients (77.4%) experienced adverse reactions during the induction phase, accounting for a total of 109 episodes. Most reactions (71.6%) were mild, resolved spontaneously, and did not require pharmacological treatment. The remaining episodes were managed with oral antihistamines and/or probiotics. No patient required intramuscular adrenaline administration, and no child discontinued treatment because of adverse events. No severe reactions according to Clark’s severity grading system were observed.

### 3.5. Immunological Parameters

#### 3.5.1. Skin Prick Test (SPT)

At baseline (T1), no statistically significant differences were observed between groups regarding SPT wheal diameter for egg yolk. However, SPT wheal diameters for egg white, OVA, and OVM were significantly higher in the OIT group compared with the historical control group (Table 1).

At follow-up (T3), the OIT group showed lower SPT wheal diameters for all evaluated egg components compared with controls. These differences reached statistical significance only for egg white (*p* = 0.0438) (Table 1).

**Figure 2:**
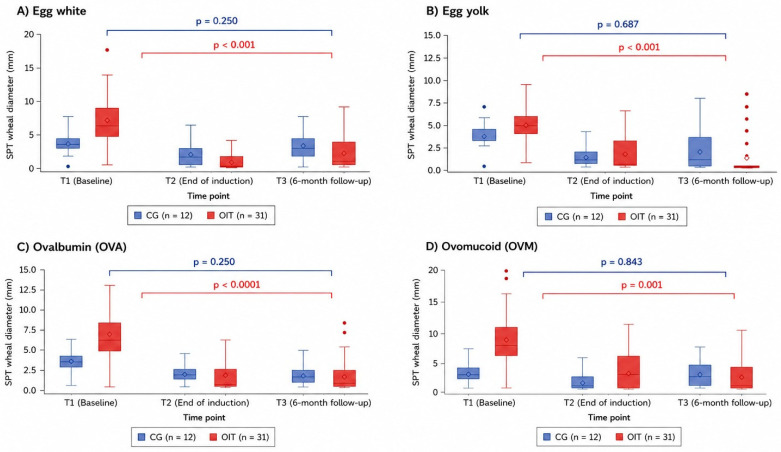
Evolution of skin prick test (SPT) wheal diameters over time in the oral immunotherapy (OIT) and historical control groups. Boxplots represent the median (line), mean (diamond), interquartile range (box), range (whiskers), and outliers (points). Blue brackets indicate within-group longitudinal comparisons in the historical control group (T1 vs. T3), whereas red brackets indicate within-group longitudinal comparisons in the OIT group (T1 vs. T3). (**A**) Egg white; (**B**) egg yolk; (**C**) ovalbumin (OVA); and (**D**) ovomucoid (OVM). *Abbreviations*: CG, historical control group; OIT, oral immunotherapy; OVA, ovalbumin; OVM, ovomucoid; SPT, skin prick test; T1, baseline assessment; T2, end of the induction phase (OIT group only); T3, six-month follow-up assessment (6 months after T2 in the OIT group or 6 months after diagnosis in the control group).

Longitudinal analysis demonstrated a significant reduction in SPT wheal diameter from T1 to T3 in the OIT group for egg white, egg yolk, OVA, and OVM (all *p* < 0.001). In contrast, no statistically significant reductions were observed in the control group for any evaluated component (Figure 2).

#### 3.5.2. Specific IgE (sIgE)

At baseline (T1), no statistically significant differences were observed between the OIT and historical control groups regarding serum sIgE levels for any of the evaluated egg components, including egg white, egg yolk, OVA, and OVM (Table 1).

At follow-up (T3), the OIT group showed significantly lower sIgE levels for all evaluated egg components compared with the control group (Table 1).

**Figure 3:**
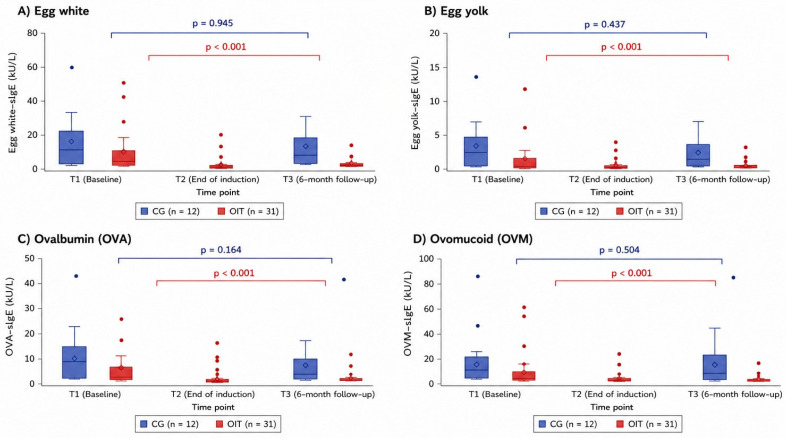
Evolution of serum-specific IgE (sIgE) levels over time in the oral immunotherapy (OIT) and historical control groups. Boxplots represent the median (line), mean (diamond), interquartile range (box), range (whiskers), and outliers (points). Blue brackets indicate within-group longitudinal comparisons in the historical control group (T1 vs. T3), whereas red brackets indicate within-group longitudinal comparisons in the OIT group (T1 vs. T3). (**A**) Egg white; (**B**) egg yolk; (**C**) ovalbumin (OVA); and (**D**) ovomucoid (OVM). *Abbreviations*: CG, historical control group; OIT, oral immunotherapy; OVA, ovalbumin; OVM, ovomucoid; sIgE, specific immunoglobulin E; T1, baseline assessment; T2, end of the induction phase (OIT group only); T3, six-month follow-up assessment (6 months after T2 in the OIT group or 6 months after diagnosis in the control group).

Longitudinal analysis demonstrated a significant reduction in sIgE levels from T1 to T3 in the OIT group for egg white, egg yolk, OVA, and OVM (all *p* < 0.001) (Figure 3).

#### 3.5.3. sIgE/Total IgE Ratio

At baseline (T1), no statistically significant differences were observed between the OIT and historical control groups regarding the sIgE/total IgE ratio for any evaluated egg component. At follow-up (T3), significant between-group differences were observed for egg white and OVM, with lower ratios in the OIT group compared with controls (Table 2).

**Figure 4:**
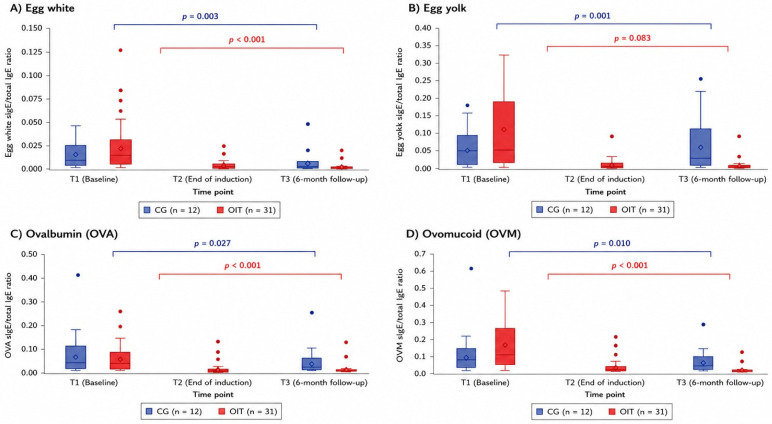
Evolution of the specific IgE/total IgE ratio over time in the oral immunotherapy (OIT) and historical control groups. Boxplots represent the median (line), mean (diamond), interquartile range (box), range (whiskers), and outliers (points). Blue brackets indicate within-group longitudinal comparisons in the historical control group (T1 vs. T3), whereas red brackets indicate within-group longitudinal comparisons in the OIT group (T1 vs. T3). (**A**) Egg white; (**B**) egg yolk; (**C**) ovalbumin (OVA); and (**D**) ovomucoid (OVM). *Abbreviations*: CG, historical control group; OIT, oral immunotherapy; IgE, immunoglobulin E; OVA, ovalbumin; OVM, ovomucoid; sIgE, specific immunoglobulin E; T1, baseline assessment; T2, end of the induction phase (OIT group only); T3, six-month follow-up assessment (6 months after T2 in the OIT group or 6 months after diagnosis in the control group).

Longitudinal analysis demonstrated a significant reduction in the sIgE/total IgE ratio from T1 to T3 in the OIT group for all evaluated egg components (all *p* < 0.001) (Figure 4).

#### 3.5.4. Specific IgG4 (sIgG4)

In the OIT group, serum-specific IgG4 (sIgG4) levels increased at the end of the induction phase (T2), followed by a slight decrease at the six-month follow-up assessment (T3). Despite this decline, sIgG4 levels remained above baseline values at T3 for all evaluated egg components. Detailed sIgG4 kinetics have been reported previously by Carrión Sari et al. [17].

sIgG4 measurements were not available for the historical control group because these determinations are not routinely performed in standard clinical practice.

### 3.6. Maintenance Phase and Six-Month Outcomes

During the six-month follow-up period, statistically significant differences were observed between the OIT and historical control groups regarding the frequency and clinical characteristics of allergic reactions (*p* = 0.0301).

#### 3.6.1. Historical Control Group

In the control group, a total of 12 allergic reactions were recorded during the six months following diagnosis, corresponding to a median of 1 reaction per patient (range: 0–2). Clinical manifestations included rhinoconjunctivitis in 1 patient (8.3%), anaphylactic reactions in 6 patients (50.0%), and cutaneous reactions in 5 patients (41.7%) (Table 1).

#### 3.6.2. OIT Group

In the OIT group, 6 patients (19.4%) experienced reactions during the maintenance phase, with one episode recorded per patient. Among these patients, four (66.7%) presented isolated cutaneous reactions (three perioral lesions and one episode of urticaria), whereas two (33.3%) experienced isolated gastrointestinal symptoms (abdominal pain and altered bowel habits). All reactions were mild and did not require treatment, OIT discontinuation, or avoidance of subsequent egg ingestion.

#### 3.6.3. Desensitization Status at T3

At the final follow-up assessment (T3), 29 of 31 patients (93.5%) in the OIT group maintained desensitization to all egg preparations, including raw, lightly cooked, and extensively cooked egg (Table 1). The remaining 2 patients (6.5%) were able to consume only extensively cooked egg products (Table 1).

These outcomes differed significantly from those observed in the control group (*p* < 0.001). At T3, 5 control patients (41.7%) remained reactive to all forms of egg, whereas the remaining 7 (58.3%) did not experience reactions after ingestion of extensively cooked egg products (Table 1). None of the patients in the control group developed tolerance to all forms of eggs during follow-up.

## 4. Discussion

### 4.1. Summary of Main Findings

This prospective controlled study demonstrates that early OIT with pasteurized egg white in children younger than two years with IgE-mediated egg allergy is highly effective and associated with a favorable safety profile. At six months of follow-up, 93.5% of treated children achieved desensitization to all egg preparations, including raw, lightly cooked, and extensively cooked egg, whereas none of the children managed with strict avoidance achieved complete tolerance during the same period.

In addition to its clinical efficacy, early OIT was associated with significant immunological changes consistent with immune modulation. These findings support the hypothesis that early intervention may promote modification of the allergic immune response during a period of increased immunological plasticity in early childhood [12].

Importantly, the safety profile observed in our cohort was favorable. Although adverse reactions during OIT were frequent, most were clinically mild and self-limited, and did not require pharmacological treatment or treatment discontinuation. No patient required intramuscular adrenaline administration. In contrast, severe reactions, including anaphylaxis, remained common in the historical control group during follow-up despite adherence to an avoidance diet. Together, these findings support the potential role of early active intervention as an alternative to passive avoidance strategies in infants with egg allergy.

### 4.2. Comparison of OIT with the Establishment of Natural Tolerance to Egg Allergens

The natural history of IgE-mediated egg allergy is characterized by gradual and heterogeneous acquisition of tolerance over time. Large longitudinal cohorts have shown that approximately 50% of affected children develop natural tolerance by 4–6 years of age, whereas a substantial proportion remain allergic into later childhood or adolescence [18,19,20].

Consistent with these observations, none of the children in our historical control group achieved complete tolerance during the six-month follow-up period, and tolerance was limited to extensively cooked egg in only 58.3% of patients. These findings reinforce the concept that spontaneous resolution of egg allergy is typically a slow process and may remain incomplete for several years.

In contrast, children receiving early oral immunotherapy achieved rapid desensitization to multiple egg preparations, including raw egg, within a relatively short period. This form of egg is generally considered more allergenic and clinically challenging than extensively heated egg products [21]. This finding may be clinically relevant because accidental exposures frequently involve incompletely cooked egg-containing foods. Longer-term studies assessing sustained unresponsiveness after oral immunotherapy are required before definitive conclusions regarding disease modification can be established. Nevertheless, previous evidence suggests that age may play a key role in the capacity to develop tolerance during food oral immunotherapy. Mechanisms related to early-life immune plasticity and enhanced responsiveness to immunomodulation may contribute to the more favorable outcomes observed when treatment is initiated during infancy [12].

### 4.3. Efficacy and Safety of Early OIT in the Context of Published Studies

The desensitization rates observed in our cohort were high, with 96.8% of patients successfully completing the induction phase and 93.5% maintaining desensitization at six months. These results compare favorably with previously published egg OIT studies performed in older pediatric populations [22]. Staden et al. [22] reported successful desensitization in 64% of children aged 0.6–12.9 years, whereas Martin-Munoz et al. [23] achieved desensitization in 84.2% of children aged 6–9 years. The higher efficacy observed in our study may be related to the younger age at intervention, which could favor immune modulation during a period of increased immunological plasticity in early life [12].

Our findings are also consistent with growing evidence suggesting that earlier initiation of food OIT may be associated with improved clinical outcomes and higher rates of sustained desensitization. Wasserman et al. [24] reported that increasing age was associated with lower treatment success and higher treatment burden in peanut OIT cohorts, supporting the rationale for intervention during early childhood.

Long-term data from food OIT studies further support the potential benefits of early intervention. In peanut allergy, Vickery et al. [25] demonstrated sustained unresponsiveness in 12 of 24 patients after discontinuation of therapy, suggesting that clinical benefits may persist beyond active treatment in a subset of individuals. Similarly, the POISED study by Chinthrajah et al. [26] showed that maintenance of desensitization after treatment withdrawal is variable and may depend on continued allergen exposure and treatment duration. In contrast, evidence regarding recurrence after naturally acquired egg tolerance remains limited. However, Leonard et al. [27] reported that regular ingestion of baked egg was associated with accelerated development of tolerance to regular egg, supporting the importance of continued dietary exposure following tolerance acquisition.

Regarding safety, adverse reactions during both induction and maintenance were predominantly clinically mild and self-limited, and no patient required intramuscular adrenaline administration. This contrasts with reports in older children, in whom systemic reactions and adrenaline use are more frequently observed during egg OIT. Vazquez-Ortiz M et al. [28] described adrenaline use in 26% of children aged 5–18 years undergoing egg OIT, whereas higher rates of severe reactions have also been reported in other cohorts involving older pediatric patients [29,30].

In contrast, the favorable safety profile observed in our cohort is consistent with studies evaluating OIT in younger children. Giavi S et al. [31] similarly reported low rates of severe adverse events in preschool-aged children, suggesting that early intervention may improve not only efficacy but also treatment tolerability.

These findings are consistent with the concept of a therapeutic “window of opportunity” for food OIT during early infancy [12,32].

Importantly, our study included a historical avoidance group, allowing direct comparison between active intervention and standard avoidance management. During follow-up, all anaphylactic reactions occurred exclusively in the control group, despite adherence to an elimination diet. Although avoidance remains the conventional standard of care, these findings highlight the limitations of avoidance strategies in preventing accidental exposures and suggest that OIT may reduce the risk of severe reactions during ongoing allergen exposure.

### 4.4. Immunological Changes

The OIT group demonstrated substantial immunological changes over the study period, including significant reductions in SPT wheal diameters, specific sIgE levels, and sIgE/total IgE ratios, together with increased sIgG4 concentrations. These findings are consistent with the immunological modifications previously described during successful food OIT [31,33,34,35].

These findings are consistent with previously described mechanisms of immune modulation during successful food OIT, including reduced Th2 activity, induction of regulatory T-cell responses, and production of blocking antibodies [36,37]. Recent mechanistic studies have further highlighted the role of allergen-specific regulatory networks, B-cell modulation, and effector-cell desensitization in the establishment of clinical tolerance during OIT [38].

Although the increase in sIgG4 levels was not statistically analyzed because of the absence of measurements in the control group, the temporal pattern observed in our cohort—an increase at the end of induction followed by partial decline at follow-up while remaining above baseline levels—is consistent with previous reports describing early immunological activation during OIT [36].

In contrast, children managed with avoidance alone showed no significant improvement in most immunological parameters during follow-up, supporting the hypothesis that passive allergen avoidance does not substantially modify the underlying allergic immune response over short-term periods.

The marked divergence in both clinical and immunological trajectories between the OIT and control groups raises the possibility that early intervention may influence the underlying disease course beyond transient desensitization [12,36]. However, longer-term studies are required to determine whether these immunological changes translate into sustained unresponsiveness and long-term disease modification.

### 4.5. Strengths and Limitations

This study has several important strengths. To our knowledge, it represents one of the few controlled comparisons between OIT and avoidance management performed exclusively in children younger than two years with IgE-mediated egg allergy. The very early age at intervention is particularly relevant given the increasing interest in exploiting the window of immunological plasticity during infancy to modify the natural course of food allergy [12].

Another major strength is the use of pasteurized liquid egg white as the OIT product. This formulation provides standardized allergen exposure, microbiological safety, reproducible protein dosing, and facilitates implementation in routine clinical practice. In addition, our study incorporated extensive immunological monitoring, including SPT, sIgE, sIgE/total IgE ratios, and sIgG4 measurements, allowing a more comprehensive characterization of the immunological changes associated with early OIT.

Importantly, the inclusion of a historical avoidance group provided a clinically meaningful comparator that is frequently lacking in pediatric OIT studies. Although not equivalent to a randomized controlled design, this comparison allowed estimation of the potential added benefit of active intervention relative to standard avoidance management.

Several limitations should also be acknowledged. First, the absence of randomization and the use of historical controls introduce the possibility of selection bias and residual confounding. Families undergoing OIT were managed at a tertiary referral center and may have been particularly motivated to pursue active treatment, potentially influencing adherence and reporting behavior. In addition, patients in the control group were recruited from a different institution and partially different recruitment periods, factors that may have introduced unmeasured clinical or environmental differences between cohorts.

Second, the sample size—particularly in the control group—was relatively small, limiting statistical power for some secondary analyses. Although exploratory multivariable analyses were considered, the limited number of participants, the small number of outcome events in the control group, and the marked imbalance in outcome distribution between groups precluded robust adjusted modeling.

Third, follow-up duration was limited to six months after completion of the induction phase. Consequently, we were unable to evaluate sustained unresponsiveness after treatment discontinuation, which remains one of the most clinically relevant long-term outcomes in food OIT studies. Furthermore, OFC was not systematically performed at T3 in the OIT group, and maintenance of desensitization was primarily assessed through clinical follow-up and parental reporting. Therefore, the reported six-month desensitization rates should be interpreted with caution, as objective confirmation by standardized OFCs was not available for all participants at the final assessment.

Finally, this was a two-center study performed within a single national healthcare context, which may limit generalizability to populations with different dietary patterns, healthcare systems, or genetic backgrounds.

### 4.6. Clinical Implications for Pediatric Practice

The findings of this study may have important implications for the clinical management of egg allergy during early childhood. Current management strategies for food allergy in children younger than two years continue to rely primarily on allergen avoidance and emergency treatment of accidental reactions. However, our results suggest that early OIT with pasteurized egg white may constitute a feasible and effective therapeutic alternative in infants with IgE-mediated egg allergy.

Early reintroduction of egg into the diet may provide benefits beyond desensitization alone. Eggs are a major source of high-quality protein and essential micronutrients, including vitamin D, iodine, folate, choline, selenium, and vitamin B12, nutrients that are particularly relevant during periods of rapid growth and neurodevelopment [6]. Therefore, prolonged elimination diets during infancy may contribute to nutritional imbalance or impaired dietary diversity in some children.

Our findings also support the emerging concept that strict allergen avoidance during early childhood should not necessarily be considered a biologically neutral strategy. Beyond the persistent risk of accidental reactions, prolonged exclusion diets may contribute to nutritional limitations [2,6], psychosocial burden [39], and delayed oral exposure during a potentially critical period of immune development [2].

In addition, food allergies impose a substantial psychosocial burden on patients and families, including anxiety related to accidental exposures, dietary restrictions, and social limitations. Early desensitization may help reduce these burdens and facilitate more normalized feeding behaviors during a critical developmental period [39]. Early intervention may also decrease the risk of persistent aversion to egg taste and texture, which has been described more frequently in older children undergoing delayed food reintroduction. Furthermore, by reducing accidental reactions and emergency healthcare utilization, early OIT may contribute to lowering the healthcare burden and associated costs of food allergy management [40].

From a practical perspective, the use of pasteurized liquid egg white may facilitate implementation of early OIT protocols by providing a readily available and easy-to-administer allergen source. These characteristics may improve feasibility in routine clinical settings. Nevertheless, despite the favorable safety profile observed in our cohort, OIT should continue to be performed in specialized allergy centers with appropriate patient selection, caregiver education, and close clinical monitoring.

Given the observational design of our study and the absence of systematic OFCs at T3, these findings should be interpreted with caution when informing clinical decision-making. However, they support consideration of early OIT within a shared decision-making framework involving clinicians and families, balancing potential benefits, treatment burden, and individual patient characteristics.

### 4.7. Future Research Directions

Further prospective studies are needed to confirm the findings of the present study and to better define the role of early OIT in the management of egg allergy during infancy. In particular, randomized controlled trials with concurrent control groups and larger sample sizes would help clarify the true magnitude of the clinical benefit and reduce the potential influence of selection bias and residual confounding inherent to observational designs.

Longer follow-up periods are also essential to determine whether early desensitization translates into sustained unresponsiveness after treatment discontinuation, which remains one of the most clinically relevant outcomes in food allergy research. Future protocols should therefore incorporate standardized OFCs after defined periods of egg avoidance to evaluate long-term immune tolerance.

Additional research should explore the broader clinical impact of early OIT, including its effects on nutritional status, growth trajectories, feeding behavior, and health-related quality of life using validated pediatric instruments. These aspects may be particularly relevant in infancy, when dietary restrictions can interfere with nutritional adequacy and psychosocial development [5].

Further immunological studies are also warranted to identify biomarkers associated with treatment response, safety, and long-term outcomes. Potential candidates include baseline sIgE levels, sIgE/total IgE ratios, component-resolved sensitization profiles, and longitudinal sIgG4 kinetics [28,36].

Finally, the optimal duration of maintenance therapy, the minimum effective maintenance dose, and the feasibility of treatment tapering or discontinuation after sustained desensitization remain important unanswered questions requiring further investigation.

## 5. Conclusions

OIT with pasteurized egg white in children younger than two years with IgE-mediated egg allergy was associated with high rates of desensitization, favorable short-term safety outcomes, and significant immunological changes consistent with immune modulation. Compared with the historical avoidance group, children receiving OIT were able to consume raw eggs and experienced fewer severe allergic reactions during follow-up. This finding may be particularly relevant for protection against accidental exposures.

These findings support the potential role of early active intervention as an alternative to exclusive avoidance strategies in infants with egg allergy. Early OIT may facilitate safer dietary diversification during a critical developmental period while reducing the clinical burden associated with persistent food allergy.

Nevertheless, given the observational design and limited follow-up duration of the present study, randomized controlled trials with longer follow-up are required to confirm long-term sustained unresponsiveness, optimize patient selection, and better define the long-term safety and effectiveness of early egg OIT.

Taken together, these findings suggest that early egg OIT performed during infancy may not only represent a desensitization strategy but also a potential opportunity to intervene during a critical phase of immune development.

## Figures and Tables

**Table 1:** Baseline and follow-up clinical, immunological, and reaction characteristics in the oral immunotherapy and control groups.

Variable	Control (*n* = 12)	OIT (*n* = 31)	*p*-Value
**Age at first reaction, months**	11.5 (6–24)	10.0 (6–15)	0.1477
**Male sex, n (%)**	9 (75.0)	14 (45.2)	0.0785
**Sensitization to other foods, n (%)**	6 (50.0)	11 (35.5)	0.4922
**Atopic dermatitis, n (%)**	9 (75.0)	15 (48.4)	0.1150
**Family history of allergy, n (%)**	6 (50.0)	15 (53.6)	0.4241
**SPT egg white at T1, mm**	3.0 (3–5)	6.0 (0–16)	0.0006
**SPT egg yolk at T1, mm**	3.0 (0–5)	4.0 (0–14)	0.0559
**SPT OVA at T1, mm**	3.0 (0–5)	7.0 (0–14)	0.0008
**SPT OVM at T1, mm**	2.5 (0–5)	8.0 (0–20)	<0.0001
**SPT egg white at T3, mm**	4.0 (0–7)	0.0 (0–9)	0.0438
**SPT egg yolk at T3, mm**	1.0 (0–7)	0.0 (0–7)	0.1303
**SPT OVA at T3, mm**	1.5 (0–4)	0.0 (0–8)	0.53
**SPT OVM at T3, mm**	2.5 (0–6)	0.0 (0–10)	0.2886
**Total IgE at T1, kU/L**	66.0 (17.7–770)	25.2 (3.94–868)	0.1369
**sIgE egg white at T1, kU/L**	6.86 (0.23–59.7)	1.95 (0.16–51.3)	0.2949
**sIgE egg yolk at T1, kU/L**	2.55 (0.04–14.5)	0.45 (0–11.9)	0.2700
**sIgE OVA at T1, kU/L**	4.01 (0.08–41.1)	0.73 (0–52.8)	0.2810
**sIgE OVM at T1, kU/L**	4.38 (0–82)	1.80 (0–55.7)	0.4832
**Total IgE at T3, kU/L**	96.0 (14.1–1420)	79.65 (3.94–3460)	0.3970
**sIgE egg white at T3, kU/L**	2.44 (0.10–73.1)	0.34 (0–7.08)	0.0102
**sIgE egg yolk at T3, kU/L**	0.38 (0–17.4)	0.00 (0–2.78)	0.0301
**sIgE OVA at T3, kU/L**	2.08 (0.05–40.1)	0.16 (0–8.68)	0.0096
**sIgE OVM at T3, kU/L**	2.99 (0.05–83.8)	0.25 (0–3.71)	0.0106
**Reactions at T1**			0.0747
Cutaneous, n (%)	10 (83.3)	13 (43.3)	
Gastrointestinal, n (%)	0	4 (13.3)	
Anaphylaxis, n (%)	2 (16.7)	13 (43.3)	
**Reactions at T3**			0.0301
Cutaneous, n (%)	5 (41.7)	4 (66.7)	
Rhinoconjunctivitis, n (%)	1 (8.3)	0	
Gastrointestinal, n (%)	0	2 (33.1)	
Anaphylaxis, n (%)	6 (50.0)	0	

Data are presented as median (range) or number (%), as appropriate. *p-values were calculated using the chi-square test, Fisher’s exact test, or Mann–Whitney U test, as indicated.* Percentages for reaction subtypes in the OIT group at T3 were calculated based on the number of patients experiencing reactions during the maintenance phase (*n* = 6). *Abbreviations*: OIT, oral immunotherapy; OVA, ovalbumin; OVM, ovomucoid; sIgE, specific immunoglobulin E; SPT, skin prick test; T1, baseline assessment; T3, six-month follow-up assessment.

**Table 2:** Evolution of the specific IgE/total IgE ratio in the oral immunotherapy (OIT) and historical control groups.

Variable	Total (*n* = 43)	Control (*n* = 12)	OIT (*n* = 31)	*p*-Value
**T1 Egg white sIgE/total IgE ratio**	0.06 (0–0.32)	0.06 (0–0.15)	0.06 (0.01–0.32)	0.345
**T1 Egg yolk sIgE/total IgE ratio**	0.01 (0–0.13)	0.01 (0–0.04)	0.01 (0–0.13)	0.575
**T1 OVA sIgE/total IgE ratio**	0.02 (0–0.33)	0.01 (0–0.33)	0.03 (0–0.22)	0.398
**T1 OVM sIgE/total IgE ratio**	0.06 (0–0.56)	0.07 (0–0.11)	0.05 (0–0.56)	0.382
**T3 Egg white sIgE/total IgE ratio**	0.01 (0–0.26)	0.03 (0–0.26)	0.00 (0–0.09)	0.012
**T3 Egg yolk sIgE/total IgE ratio**	0.00 (0–0.04)	0.00 (0–0.04)	0.00 (0–0.01)	0.298
**T3 OVA sIgE/total IgE ratio**	0.00 (0–0.23)	0.01 (0–0.23)	0.00 (0–0.09)	0.147
**T3 OVM sIgE/total IgE ratio**	0.00 (0–0.18)	0.03 (0–0.18)	0.00 (0–0.03)	0.001

Data are presented as median (range). *p*-values correspond to between-group comparisons performed using the Mann–Whitney U test. *Abbreviations*: OIT, oral immunotherapy; OVA, ovalbumin; OVM, ovomucoid; sIgE, specific immunoglobulin E; IgE, immunoglobulin E; T1, baseline assessment; T3, six-month follow-up assessment.

## Data Availability

The data supporting the findings of this study are available from the corresponding author upon reasonable request. Extracted study data used for the meta-analysis are included within the manuscript and Supplementary Materials.

## Supplementary Materials

The following supporting information can be downloaded at: https://www.mdpi.com/article/10.3390/children13060810/s1, Table S1: Oral immunotherapy protocol with pasteurized egg white; Figure S1: STROBE flow diagram of participant selection and follow-up; File S1: STROBE Checklist for the Reporting of Observational Studies; File S2: Ethics Committee approval issued by the Research Ethics Committee of the Autonomous Community of Aragon; File S3: Ethical approval acknowledgement issued by the Drug Research Ethics Committee of Burgos and Soria.

## Abbreviations

The following abbreviations are used in this manuscript:

IQR	Interquartile range
OFC	Oral food challenge
OIT	Oral immunotherapy
OVA	Ovalbumin
OVM	Ovomucoid
SD	Standard deviation
sIgE	Specific immunoglobulin E
sIgG4	Specific immunoglobulin G4
SPT	Skin prick test
STROBE	Strengthening the Reporting of Observational Studies in Epidemiology
T1	Baseline assessment
