# Supplementary material for: Early Oral Immunotherapy with Pasteurized Egg White in Children Younger than Two Years with IgE-Mediated Egg Allergy: A Prospective Study with Historical Controls
Source: Children (Basel). 2026 Jun 12;13(6):810. doi: 10.3390/children13060810 (PMC13298391; doi:10.3390/children13060810)
Supplement: Supplementary file 1 [file children-13-00810-s001.zip › children-4366306-supplementary.pdf]

Table S1. Oral immunotherapy protocol with pasteurized egg white.

Week	Dilution	Dose (mL)	Egg protein dose (mg)
1	1:1	0.1	11
2	1:1	0.2	22
3	1:1	0.4	44
4	1:1	0.7	77
5	1:1	1.0	110
6	1:1	1.3	143
7	1:1	2.0	220
8	1:1	2.5	275
9	1:1	3.2	352
10	1:1	4.0	440
11	1:1	5.0	550
12	1:1	6.2	682
13	1:1	8.0	880
14	1:1	11.0	1210
15	1:1	15.0	1650
16	1:1	22.0	2475
17	1:1	30.0	3300

Supporting Information Table S1. Weekly dose-escalation protocol used for oral immunotherapy with pasteurized liquid egg white. Dose increases were performed under hospital supervision, whereas the same dose administered during the previous hospital visit was given daily at home between visits.

Figure S1. STROBE flow diagram of participant selection and follow-up.

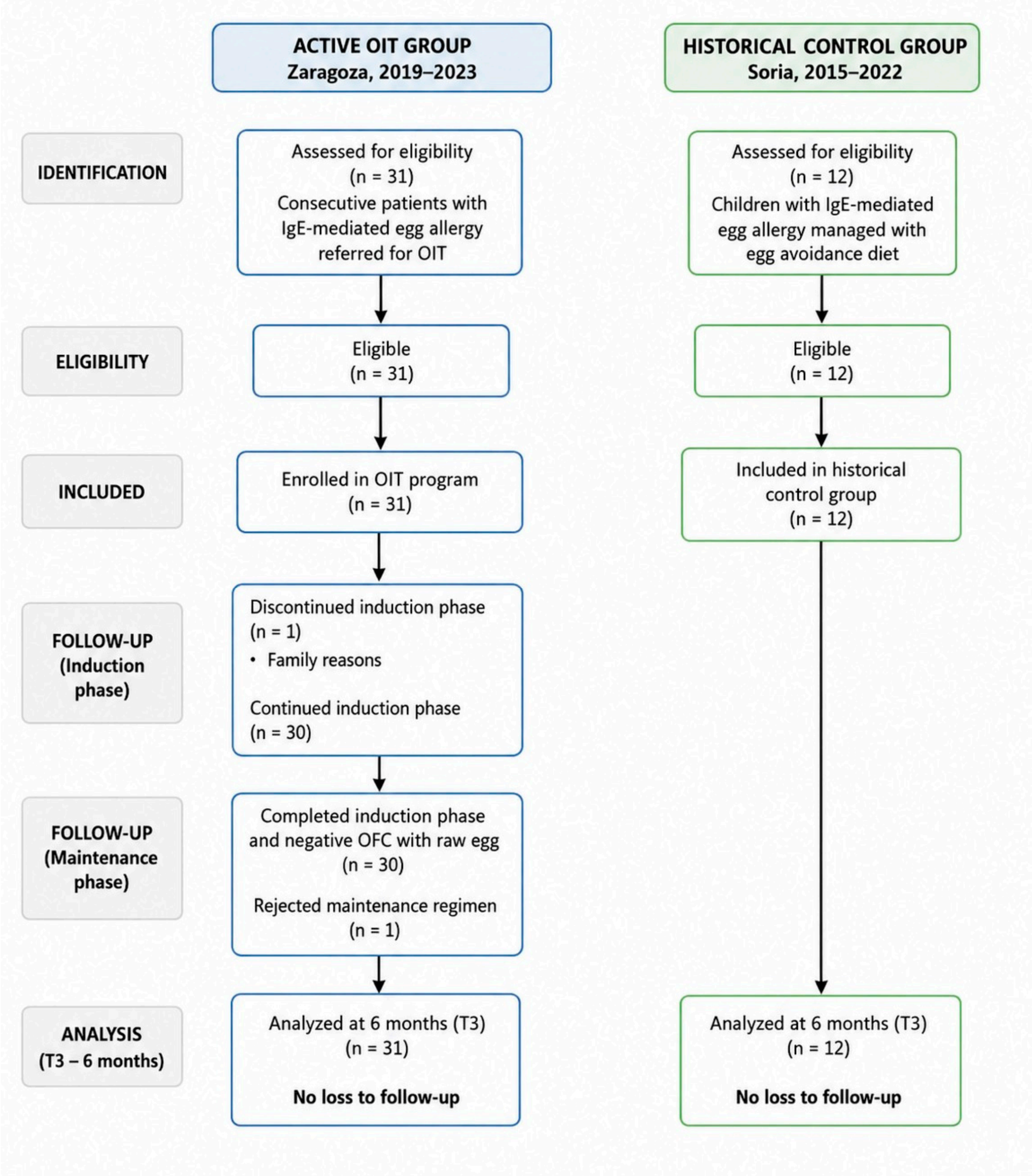

Supplementary File S1. STROBE Checklist for the reporting of observational studies.

Section/Item	Recommendation	Reported on page No.
**Title and abstract**		
1(a)	Indicate the study's design with a commonly used term in the title or the abstract	Title page, Abstract
1(b)	Provide in the abstract an informative and balanced summary of what was done and what was found	Abstract
**Introduction**		
2	Explain the scientific background and rationale for the investigation being reported	Introduction
3	State specific objectives, including any prespecified hypotheses	End of Introduction
**Methods**		
4	Present key elements of study design early in the paper	Section 2.1
5	Describe the setting, locations, and relevant dates	Section 2.2
6(a)	Give the eligibility criteria and sources and methods of participant selection	Sections 2.2–2.3
7	Clearly define all outcomes, exposures, predictors, confounders, and effect modifiers	Sections 2.4–2.7
8	For each variable of interest, give sources of data and details of methods of assessment	Sections 2.4–2.7
9	Describe any efforts to address potential sources of bias	Sections 2.1 and 4.5
10	Explain how the study size was arrived at	Section 2.2
11	Explain how quantitative variables were handled in the analyses	Section 2.8
12(a)	Describe all statistical methods	Section 2.8
12(b)	Describe methods used to examine subgroups and interactions	Not applicable
12(c)	Explain how missing data were addressed	Section 2.8
12(d)	If applicable, explain how loss to follow-up was addressed	Not applicable
12(e)	Describe any sensitivity analyses	Not applicable
**Results**		
13(a)	Report numbers of individuals at each stage of the study	Figure S1, Results
13(b)	Give reasons for non-participation at each stage	Results
13(c)	Consider use of a flow diagram	Figure S1
14(a)	Give characteristics of study participants	Table 1
14(b)	Indicate number of participants with missing data for each variable	Results
15	Report numbers of outcome events or summary measures	Results
16(a)	Give unadjusted estimates and, if applicable, confounder-adjusted estimates	Results

Section/Item	Recommendation	Reported on page No.
16(b)	Report category boundaries when continuous variables were categorized	Not applicable
16(c)	If relevant, consider translating estimates of relative risk into absolute risk	Not applicable
17	Report other analyses done	Results
**Discussion**		
18	Summarize key results with reference to study objectives	Discussion
19	Discuss limitations of the study	Section 4.5
20	Give a cautious overall interpretation of results	Sections 4.1–4.6
21	Discuss the generalizability of the results	Sections 4.5–4.6
**Other information**		
22	Give the source of funding and the role of funders	Funding Statement

Abbreviations: OIT, oral immunotherapy; OVA, ovalbumin; OVM, ovomucoid; sIgE, specific immunoglobulin E; sIgG4, specific immunoglobulin G4; SPT, skin prick test.

Supplementary File S2. Ethics Committee approval issued by the Research Ethics Committee of the Autonomous Community of Aragon (CEICA; protocol code PI19/315).

GOBIERNO DE ARAGON
Departamento de Sanidad

Informe Dictamen Favorable
Trabajos académicos
C.P. - C.I. PI19/315
24 de julio de 2019

Dña. María González Hinjos, Secretaria del CEIC Aragón (CEICA)

CERTIFICA

1º. Que el CEIC Aragón (CEICA) en su reunión del día 24/07/2019, Acta Nº 14/2019 ha evaluado la propuesta del Trabajo:

Título: Protocolo de Inmunoterapia oral con clara pasteurizada en pacientes menores de dos años con alergia a proteínas del huevo

Alumna: Silvia Karina Carrión Sari
Directores: Carlos Colás y Mª Teresa Sobrevía Elfau

Versión protocolo: v1.2, 17/07/2019
Versión documento de información y consentimiento: v1.2, 17/07/2019

2º. Considera que

- El proyecto se plantea siguiendo los requisitos de la Ley 14/2007, de 3 de julio, de Investigación Biomédica y los principios éticos aplicables.
- El Tutor/Director garantiza la confidencialidad de la información, la correcta obtención del consentimiento informado, el adecuado tratamiento de los datos en cumplimiento de la legislación vigente y la correcta utilización de los recursos materiales necesarios para su realización.

3º. Por lo que este CEIC emite **DICTAMEN FAVORABLE a la realización del proyecto**.

Lo que firmo en Zaragoza

GONZALEZ HINJOS MARIA - DNI 03857456B
Firmado digitalmente por GONZALEZ HINJOS MARIA - DNI 03857456B
Fecha: 2019.07.29 08:54:39 +02'00'

María González Hinjos
Secretaria del CEIC Aragón (CEICA)

Página 1 de 1

Tel. 976 71 5836 Fax. 976 71 55 54 Correo electrónico mgonzalezh.ceic@aragon.es

Supplementary File S3. Ethical approval acknowledgement issued by the Drug Research Ethics Committee of Burgos and Soria (CEIm Burgos-Soria; reference number 2880).

Complejo Asistencial Universitario de Burgos
Avda. Islas Baleares, 3 - 09006 BURGOS
Teléfono 947 28 18 00

Sacyl

Informe del Comité de Ética de la Investigación con Medicamentos

Don Jorge Labrador Gómez Secretario Técnico del Comité de Ética de Investigación con Medicamentos del Área de Salud Burgos y Soria,

CERTIFICA:

Que este Comité ha informado de la propuesta para que se realice el Estudio, titulado: **"Evolución clínica y analítica de los niños menores de 36 meses con alergia a proteínas del huevo en el Hospital Santa Bárbara. Soria"** (*Ref. CEIm 2880)* para que sea realizado por la Dra. Silvia Karina Carrión Sari del Servicio de Alergología del Hospital Santa Bárbara de Soria como investigador principal.

Este comité constata qué a dicho Estudio, no le es de aplicación el Real Decreto 1090/2015 de Ensayos Clínicos con Medicamentos. Este CEIm de Área de Salud de Burgos y Soria se da por enterado.

Lo que firmo en Burgos, 28 de marzo de 2023

D. Jorge Labrador Gómez

Junta de Castilla y León
Consejería de Sanidad

HG-378
